# Developmental Trends in the Application and Measurement of the Bidirectional Reflection Distribution Function

**DOI:** 10.3390/s22051739

**Published:** 2022-02-23

**Authors:** Yangyang Zou, Liu Zhang, Jian Zhang, Bonan Li, Xueying Lv

**Affiliations:** 1 College of Instrumentation and Electrical Engineering, Jilin University, Changchun 130012, China; zouyy19@mails.jlu.edu.cn (Y.Z.); libn20@mails.jlu.edu.cn (B.L.); lvxueying@jlu.edu.cn (X.L.); 2 School of Opto-Electronic Engineering, Changchun University of Science and Technology, Changchun 130022, China; zhangjian_nr@126.com

**Keywords:** BRDF, traditional measurement, fast measurement, developmental trends

## Abstract

The bidirectional reflection distribution function (BRDF) is among the most effective means to study the phenomenon of light–object interaction. It can precisely describe the characteristics of spatial reflection of the target surface, and has been applied to aerial remote sensing, imaging technology, materials analysis, and computer rendering technology. This study provides a comprehensive review of the development of devices to measure the BRDF. We gathered research in the area by using the Web of Science Core Collection, and show that work on the BDRF has been ongoing in the last 30 years. We also describe some typical measurement devices for the BRDF proposed in the literature. Finally, we summarise outstanding problems related to BRDF measurement and propose directions of future research in the area.

## 1. Introduction

The interaction between electromagnetic waves and the surface of objects consists of three processes: reflection, absorption, and transmission. The reflection of electromagnetic waves by the object is related to the roughness of its surface and the wavelength of the waves. The surfaces of all objects in nature are neither ideally smooth, like a mirror, nor ideally Lambertian. Reflection in these cases cannot be described simply by specular reflection or diffuse reflection, but by the scattering of light with both specular and diffuse components in hemispheric space. Nicodemus proposed the bidirectional reflectance distribution function (BRDF) in 1965 to characterize the properties of spatial reflectance of the target surface [1]. The BRDF combines specular and diffuse reflections to provide a more realistic and accurate description of the characteristics of spatial reflection off the surface of the target object.

**Figure 1:**
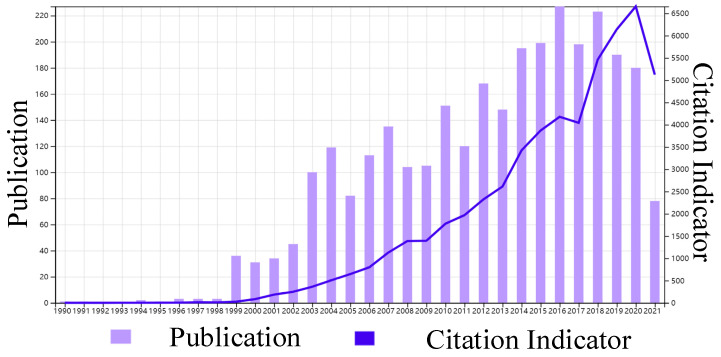
Number and citation frequency of BRDF-related papers in English.

According to the Web of Science (WOS), a total of 2995 papers related to the BDRF were published between 1 January 1990 and 30 June 2021, and were cited up to 25,877 times. Figure 1 shows the number and frequency of citation of the relevant literature in English as indexed in the WOS with “BRDF” as the keyword. It shows that studies on the BRDF have increased in number in the last three decades.

**Figure 2:**
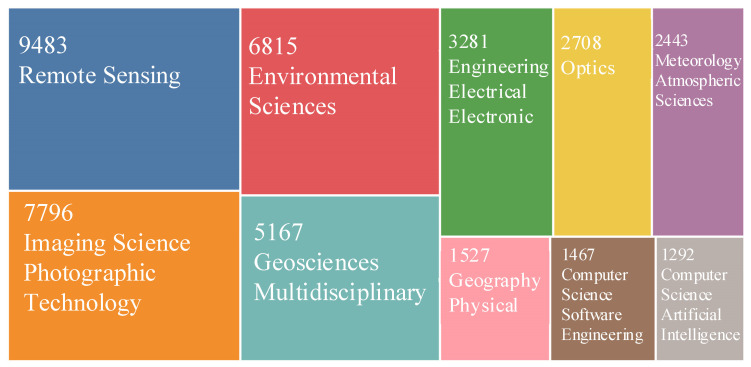
Citations of the BRDF according to the WOS Core Collection.

An analysis of the cited literature in the WOS Core Collection is shown in Figure 2 It reveals that the BRDF covers a wide range of fields, such as remote sensing, environmental science, imaging technology, optics, and computing.

In remote sensing, the calculation of surface albedo refers to the ratio of the total reflected light flux in each direction to the total incident light flux. The surface Lambeau hypothesis leads to a 45% error in albedo calculations [2], Stroeve et al. [3] found in their study that the inversion accuracy of illumination could be improved by combining spectral data with multi-angle BRDF data. Currently, landmark albedo products in-orbit, such as POLDER, MISR, MOIDS, and MERIS [4,5,6,7], are estimated based on surface bidirectional reflection characteristics.

In environment and Earth science, vegetation canopy structure parameters are key input parameters of ecosystem productivity models, global climate, and hydrological models. The surface reflectance of different vegetation is anisotropic due to its structural distribution. For example, BRDF distribution tests on wheat leaves [8], sorghum [9], maple leaves, and other leaves [10] can be found that different vegetation has different BRDF characteristic models. Therefore, based on the sensitivity of multi-angle BRDF data observation to vegetation structure, BRDF data can improve the accuracy of vegetation classification to 91% [11], which can improve the assessment of the ecological environment in different regions.

In optical research, stray light is a non-negligible aspect of all optical design, and its suppression is the basis for obtaining high-quality images. BRDF/BTDF (Bi-directional Transmittance Distribution Function) data can be used to quantify the scattering characteristics, and BRDF/BTDF spatial distribution data on the surface of optical elements can be used in stray light modeling in FRED, ASAP, Zemax, and other optical software [12,13,14]. As for stray light suppression materials required in some space optical systems, their inhibitory effect on stray light can also be judged by analyzing their BRDF data [15,16].

In computer model rendering and imaging, performance in terms of processing the microstructure of a given surface based on BRDF data can be used to determine the degree of ‘realism’ in areas such as special effects and 3D animation. As visual attributes, gloss and texture are the physical information of BRDF distribution in hemispherical space [17,18,19]. The authenticity of human skin in animation production can be realized through a large amount of BRDF model data. In 2006, T. Weyrich et al. [20] measured and estimated skin BRDF data of people of different genders and races. L. Hanssen et al. [21] and G.S. Won et al. [22] also carried out a lot of work on the establishment of BRDF data of human skin, providing data support for rendering technology of the human model.

The use and calibration of basic measurement devices in meteorology, such as the Transmission Visibility Meter (TVM) and the Forward Scattering Visibility Meter (FSVM), are based on atmospheric scattering characteristics. The calibration of TVM is achieved by using the scattering characteristics of standard scatterers [23,24], while the FSVM measures atmospheric visibility by measuring the scattering coefficient in the fixed direction of the atmosphere [25,26].

**Figure 3:**
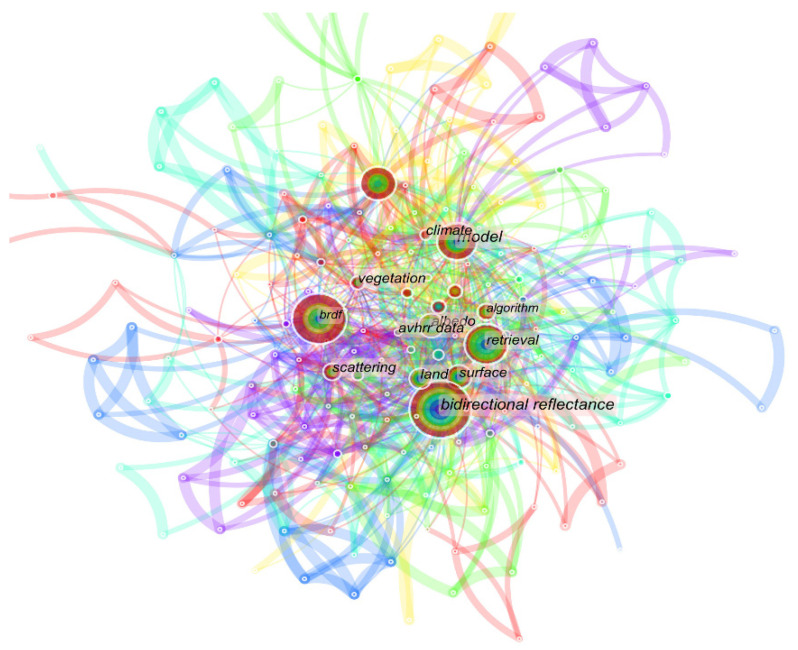
CiteSpace software-based co-occurrence network diagram of BRDF keywords.

**Figure 4:**
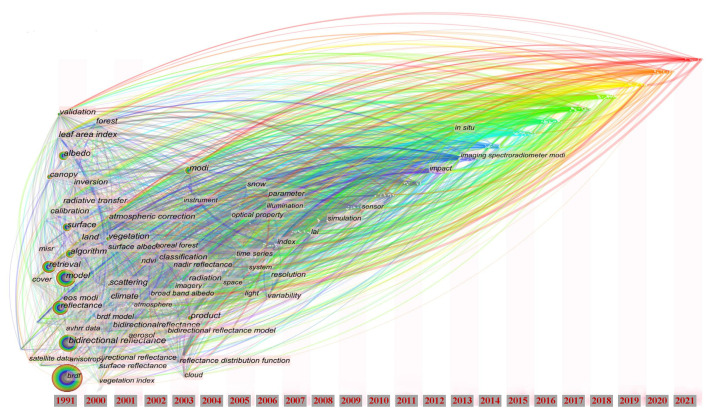
Results of analysis of the timeline of research on BRDF based on CiteSpace software (School of Information Science and Technology, Drexel University, USA).

An analysis of the literature retrieved from the WOS Core Collection using CiteSpace software, shown in Figure 3, reveals that BRDF-based model building, imaging techniques, surface characterization, and radiation models are popular issues in the relevant research. The statistics based on a timeline shown in Figure 4 also reveal that research on the BRDF has been ongoing for three decades.

In summary, accurate measurements are a prerequisite for appropriate application. This paper reviews the developmental history of BRDF measurement devices and discusses typical BRDF measurement structures. The aim is to summarise outstanding problems and propose topics for future research.

## 2. History of Developments in BRDF Measurements

Measurements are the basis for the application of bidirectional reflectance distribution functions. Depending on the characteristics of the target and the needs of modelling, measurements of the target object are generally required to cover the characteristics of the BRDF in a specific spatial angular range centred on the sample.

**Figure 5:**
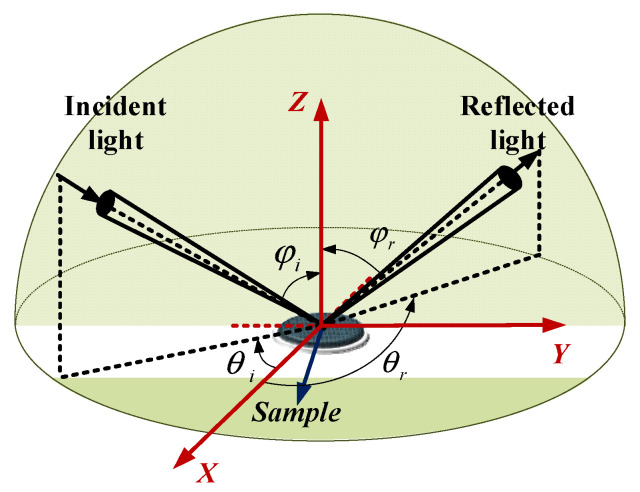
Spatial distribution diagram of BRDF hemisphere.

Since Nicodemus proposed a definition of the BDRF in 1965 [1], various measurement devices have been designed in recent decades to measure the BRDF of different targets. The BRDF is a spatial function of four dimensions: the incident zenith angle $\theta_{i}$ the incident azimuth angle $\varphi_{i}$, the reflected zenith angle $\theta_{r}$ and the reflected azimuth angle $\varphi_{r}$ (as shown in Figure 5). BRDF is defined as the ratio of scattering illuminance to incident irradiance of the light source,

(1) $$f(\theta_{i},\varphi_{i},\theta_{r},\varphi_{r},\lambda )=\frac{dL_{r}(\theta_{i},\varphi_{i},\theta_{r},\varphi_{r},\lambda )}{dE_{i}(\theta_{i},\varphi_{i})}.$$

Its measurement system is, thus, mainly determined by the spatial relationship among the light source, the sample, and the detector as well as the processing of the detected data. Bartell et al. [27] made a detailed analysis of the definition and measurement of BRDF, and pointed out that in the process of BRDF measurement, the size of the sample, the size of the beam, and the effective receiving area of the detector are all problems that need to be paid attention to in actual measurement. In addition, from an optical point of view, it is necessary to consider the type of light source, such as polarization or not, and the speckle on the sample surface will affect the final test signal. G. Meister et al. [28,29] also analyzed the measurement error of BRDF.

**Figure 6:**
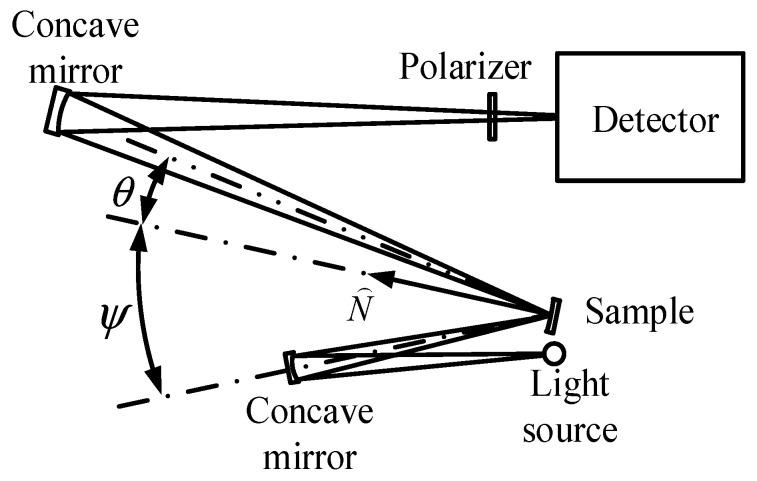
Schematic diagram of BRDF measurement system. Reprinted with permission [30]. Copyright OSA, 1966.

The first BRDF measurement device was developed in 1966 by Sparrow, E.M. et al., as shown in Figure 6. The test system mainly includes a light source, a sample, two concave reflectors, a polarizer, a monochromator, and a detector. Two corner devices control the light source and the turntable on which the sample is placed so that both can perform one-dimensional (1D) rotation in the plane. The main purpose of this measurement device is to study the effect of such factors as the direction of the incident light and the roughness of the surface on the BDRFs of magnesium oxide ceramics and aluminium-coated frosted glass [30]. The detector in this test system is fixed, and the motion of the light source and the sample can be used to obtain 2D BRDF data (incident and reflected zenith angles) in a 1D plane, where the structure is relatively simple and rough.

**Figure 7:**
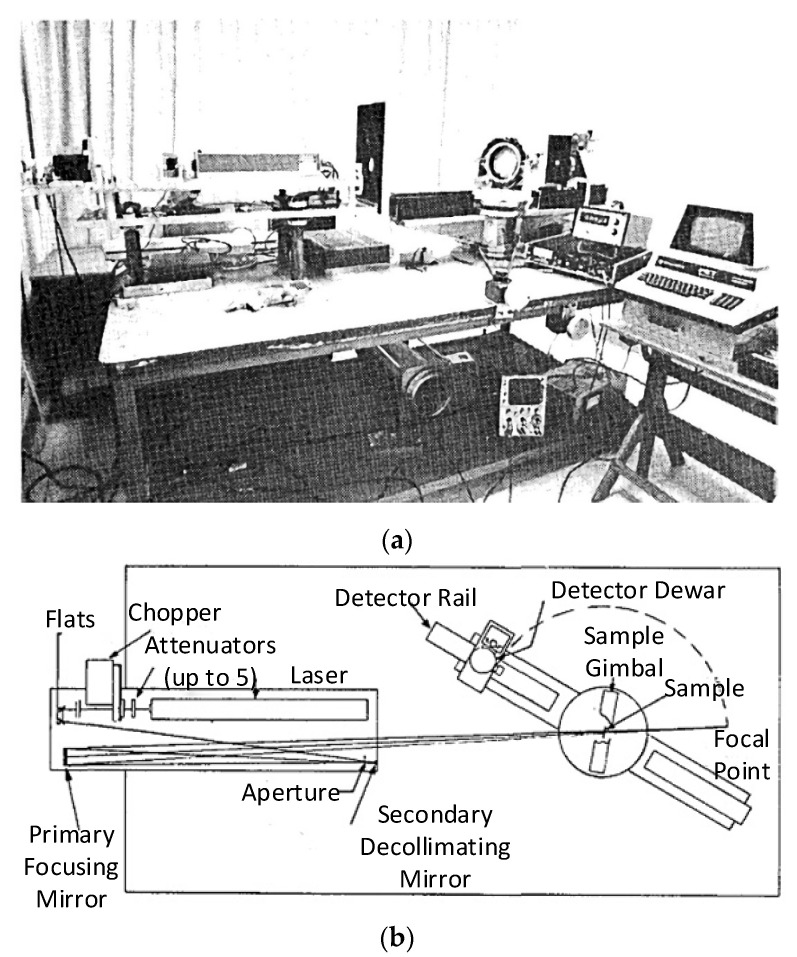
Equipment to measure scattering equipment and its layout. (**a**) Measurement system physical diagram; (**b**) Measurement system schematic diagram. Reprinted with permission [31]. Copyright OSA, 1981.

To investigate the characteristics of reflection of gold-plated sandpaper at different wavelengths and incidence angles, a device to measure scatterings developed by the Arizona Center for Optical Sciences, and used by Stuhlinger et al., at the University of Arizona, in 1980, is shown in Figure 7. The detector in this system is mounted on a kinematic mechanical arm rotating in the horizontal plane. The amount of radiation scattered from the sample surface is determined, and the characteristics of scattering of the sample at different angles of incidence are obtained by rotating the sample along the vertical axis of its centre [31]. The light source in the test system is fixed, and 2D BRDF (incident and reflected zenith angles) data are measured on the sample surface by rotating it and the detector. The source of laser light used by the system features three wavelengths, 0.6328 μm, 3.39 μm, and 10.6 μm, and each requires a different detector.

**Figure 8:**
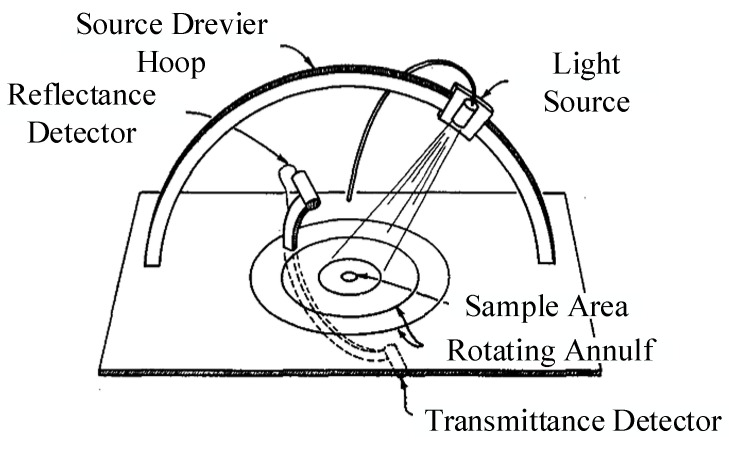
Bi-directional goniometer. Reprinted with permission [32]. Copyright Taylor and Francis, 1990.

According to the definition of the BRDF, its measurement should be carried out along four dimensions. In 1990, Murray-Coleman and Smith designed a set of bidirectional reflectometers for BRDF measurement based on the circular orbital structure of the zenith as shown in Figure 8. The light source, detector, and sample are fixed on a stepper motor, and the BRDF of the material is measured by moving the light source, sample, and detector relative to one another through motor control software. The system provides four degrees of freedom. The light source can be used to adjust the range of the zenith angle to ±85.6° and the azimuth angle to 360°. The detector can be used to adjust the range of the zenith angle to 0°~78.3° and the azimuth angle to 360°. The BRDFs of different materials were measured using this system, and the authors concluded that the size of the light source was dependent on the sample size, and spatial resolution can be improved by reducing the magnitude of irradiation from the light source, and, thus, the size of the sample measurements [32]. Although the system gives the design concept of four degrees of freedom measurement, but the measurement only for isotropic sample surface actually, the final result is only the measurement of incident zenith angle and reflection zenith angle. However, the system can measure both the bi-directional reflection distribution function and bi-directional transmission distribution function of the sample by moving the detector in the zenith angle space range. The measurement system is a typical BRDF mechanical structure—the arc orbit goniometer structure, which is the reference for the design of a large number of subsequent conventional BRDF measurement structures.

**Figure 9:**
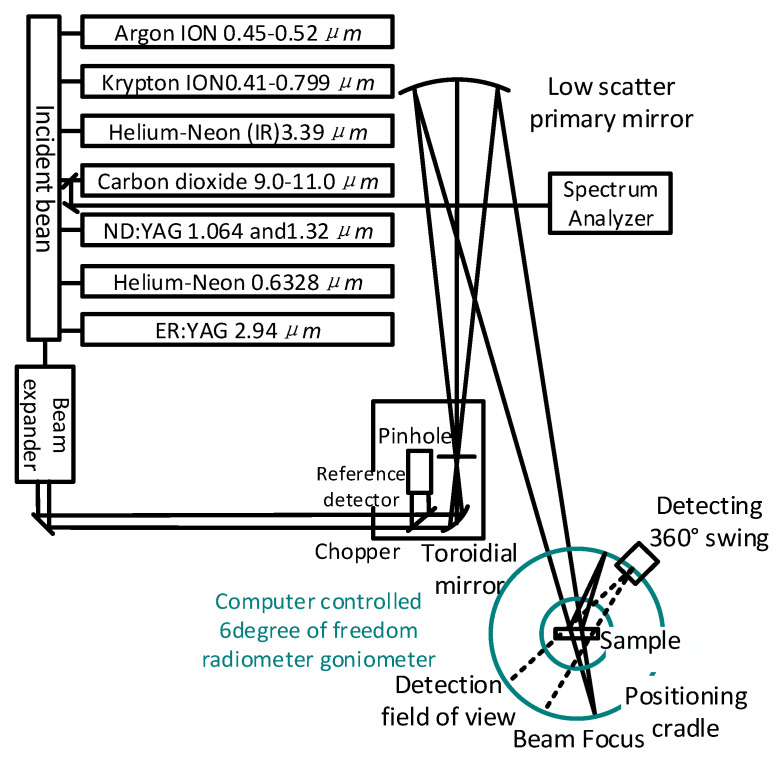
Configuration schematic of multi-wavelength BRDF measurement system. Reprinted with permission [33]. Copyright ARC, 1992.

To study the model to analyse surface reflectance based on multiple light sources, a greater variety of wavelengths of light need to be fused to obtain integrated surface reflectance. A BRDF measurement system containing light sources of multiple wavelengths was thus designed and built by Drolen, of Houston Aviation, in 1992, and is shown in Figure 9. The incident light source of the system consisted of six selectable lasers, and the radiation goniometer was mounted on a four-axis structure controlled by four stepper motor drives. By controlling the motor to convert the coordinates of the goniometer into those of the position of the sample as defined by the BRDF, the incident azimuth angle of the light source was kept at 0°, the incident zenith angles were five angles uniformly selected by cosine integration in the range of 5° near normal incidence to 78° near swept incidence, the detected azimuth angles were at eight angular positions equally divided in the range of 0°~180°, and the zenith angle consisted of eight angles obtained by cosine integration in the range of 0°~180°. Several materials used in spacecraft were measured using this system: the BRDF of black Kapton @0.488 μm, the BRDF of indium tin oxide (ITO)/Kapton/Al @0.488 μm, and the BRDF of Chemglaze white paint @0.488 and 10.63 μm. The multi-spectral BRDF measurement data were combined to calculate the high luminosity of surfaces of commonly used in spacecraft materials [33].

**Figure 10:**
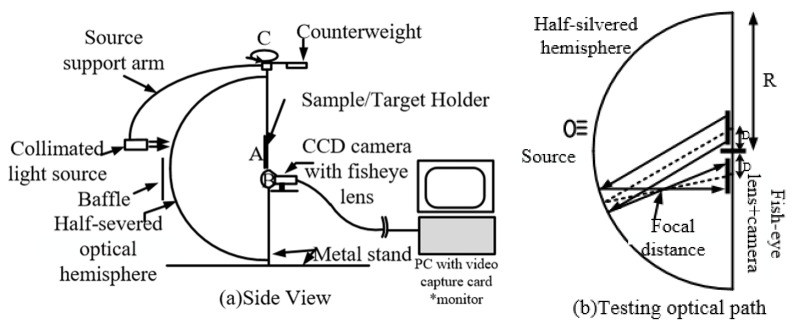
LBL imaging goniometer. Reprinted with permission [34]. Copyright ACM, 1992.

The accurate measurement of BRDF data on anisotropic surfaces is required to obtain the spatial distribution of the material in the hemisphere (reflected zenith angle and reflected azimuth). In 1992, Gregory J. Ward, of the Lawrence Berkeley Laboratories (LBL), designed a goniometer based on imaging technology, as shown in Figure 10. In view of the complex structure of previous goniometers, which require a long time to measure the BRDF distribution of an anisotropic surface and are expensive, he developed a simpler measurement device, the LBL imaging goniometer. The system consisted of a silver-plated hemisphere as key optical device and a Charge Coupled Device (CCD) camera with a fisheye lens. Based on the traditional goniometer, the mechanical structure controlled two degrees of freedom of the light source, combined with the hemisphere structure and the fisheye lens, to form the LBL imaging goniometer. The light source was irradiated to the sample placed at point A. The light reflected from the sample surface was collected by the hemispherical mirror and reflected to the fisheye lens and the CCD. Anisotropic BRDF data for aluminium materials at several incidence angles were obtained by this measurement system and were applied to the rendering technique [34]. The system is restricted by the size of the reflective hemisphere, its shape, its material, and the sample size. The BRDF testing capability near the swept angle of incidence of the sample was limited, while the accuracy of the hemisphere and the collimation of the light source were problems for the system as well such that the final results were inaccurate. However, this was the first system to use optical means to simplify the mechanical structure, and it used an imaging technique to measure the BRDF on the surface of anisotropic materials.

**Figure 11:**
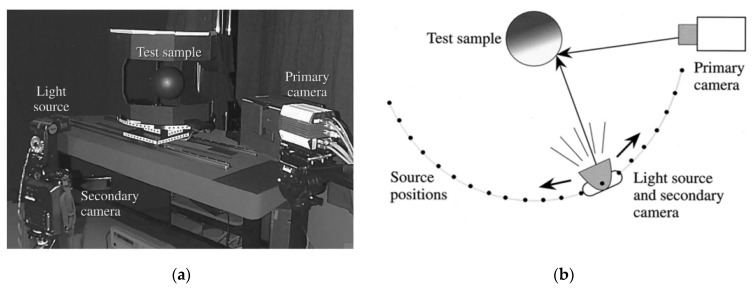
Image-based BRDF detection. (**a**) Measurement system physical diagram; (**b**) Measurement system schematic diagram. Reprinted with permission [35]. Copyright OSA, 2000.

Given that imaging technology can be applied to BRDF data measurement, Stephen et al., of Cornell University, designed a BRDF measurement device based on imaging technology in 1999, as shown in Figure 11. The aim was to simulate computer-synthesised scenes by approximating images of realistic scenes to establish a complete model of the reflective surface. The measurement system consisted of a camera (12-bit CCD still camera), a light source (Nikon SB-16), and a secondary camera (DCS420). The primary camera was fixed to test the reflected brightness of the sample, and the secondary camera was assembled with the light source to determine its position after it had been moved by using automatic photogrammetry and 3D positional relationships. The test system took a series of photographs of a bent sample, which contained partial information on the light reflected from the surface in different directions, and then analysed them according to the shape of the sample shape and the position of the light source to obtain its BRDF. The BRDF measurement technique based on imaging technology can quickly acquire the BRDF of the sample surface, and the test range can help cover the entire hemispheric space close to the swept incidence angle. This can be used to establish a large sample database of the BRDF, but the technique is limited to the measurement of curved objects with a uniform distribution of the isotropic BRDF [35].

**Figure 12:**
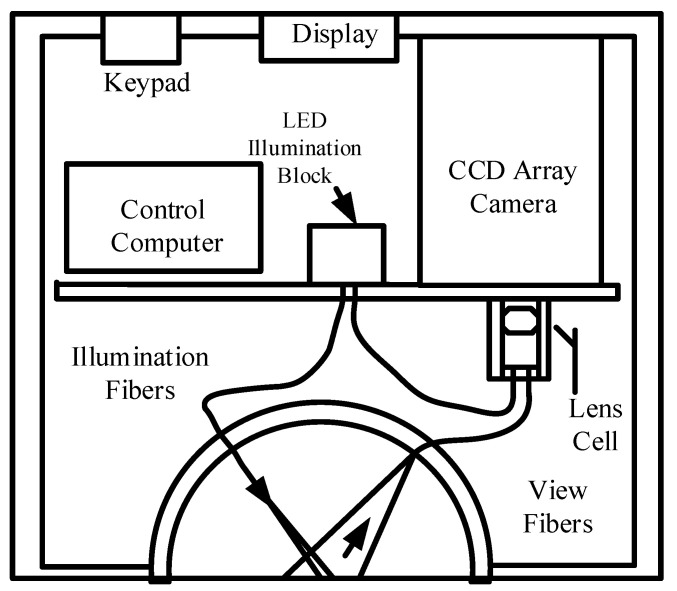
BRDF detection system based on fiber-optic technology. Reprinted with permission [36]. Copyright OSA, 2000.

In view of the complex structure of the BRDF measurement machinery, it can be used only indoors to measure various materials. To enable the use of the equipment in the field to measure the BRDF of wet sand surfaces under water, and compare it with that of dry sand surfaces, Yu et al., of the University of Miami, applied optical fiber to an incident light source for BRDF detection at eight illumination angles in the range of 0°~65°, three spectral bands of 475 nm, 570 nm, and 658 nm, and within the ranges of 5°~65° of the zenith angle and 0°~±180° of the azimuth angle. The main devices of the detection system are shown in Figure 12. Eight angles of incidence (0°, 5°, 15°, 25°, 35°, 45°, 55°, and 65°) were used with LED as light source, and optical fibers were used to direct the light to a spherical lens for collimation and irradiation onto the sample surface. The directions of detection were mainly specular and backward. Holes were drilled in an aluminium dome at zenith angles of 5°, 15°, 25°, 35°, 45°, 55°, and 65°, and azimuth angles of ±5°, ±10°, ±15°, ±30°, ±45°, ±60°, ±75°, ±90°, ±105°, ±120°, ±135°, ±150°, ±165°, ±172°, and ±180° to access the observation fiber. The observation fiber received scattered light from the sample surface and transmitted it to a CCD camera to measure the BRDF. The instrument was used to help determine the surface characteristics of the seafloor. The complex structure of mechanical movement was abandoned, and a simple, compact, and portable structure was designed to measure BRDF data using optical fibers [36].

**Figure 13:**
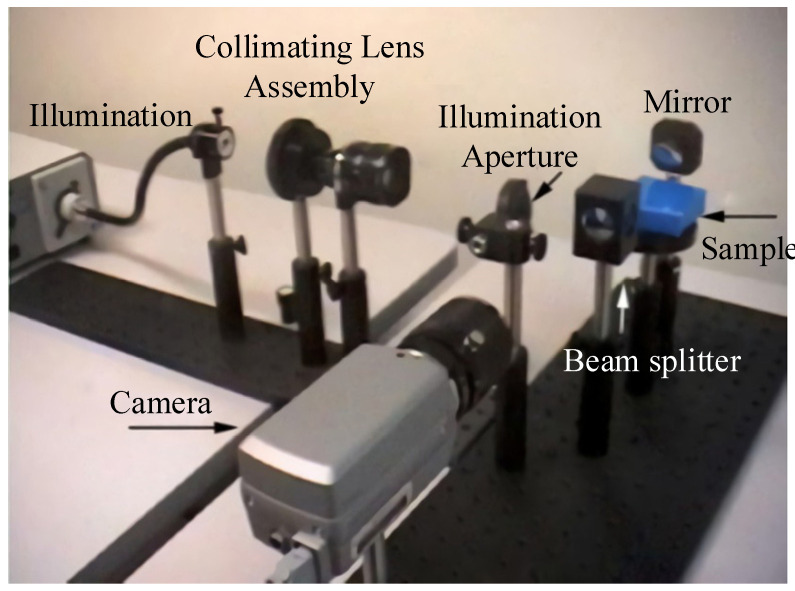
Parabolic experimental system for BRDF detection. Reprinted with permission [37]. Copyright IEEE, 2001.

To further improve the measurement efficiency of the system based on a compact structure, Kristin J. Dana, of Rutgers University, proposed a fast BRDF measurement system based on aspheric optical characteristics, as shown in Figure 13. The device mainly consisted of a light source, a collimating lens, an illumination (incident) aperture, a beam splitter, an off-axis parabolic mirror, and a camera. By moving the illumination aperture to achieve the incidence of light on the surface of the sample at different angles, this method can be used to translate the complex movement of the light source of the goniometer into the illumination aperture. The incident light irradiated to the sample surface produces scattered light collected by the off-axis parabolic mirror, the surface points of which are imaged by the CCD camera. The BRDF distribution on the surface of the skin can be acquired using this system. The system eliminates the need for complex mechanical equipment to move the light source and the detector within the hemisphere, and is convenient and fast [37].

**Figure 14:**
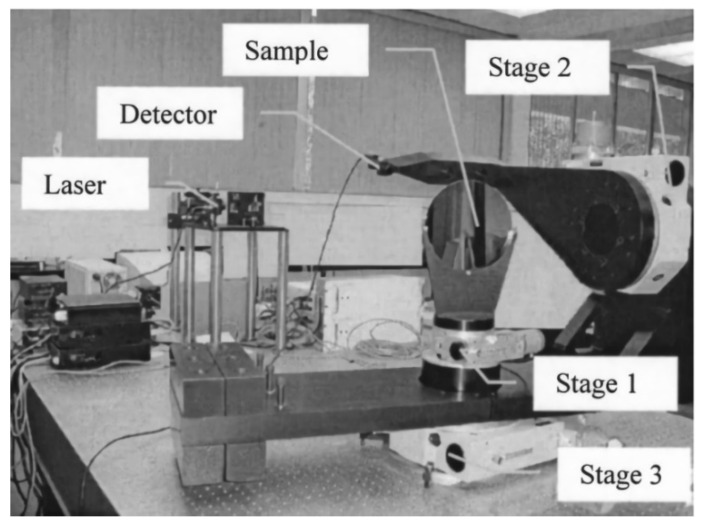
TAAS scatterometer. Reprinted with permission [38]. Copyright AIP, 2003.

While devices for rapid BRDF measurement based on optical characteristics were being proposed, those based on mechanical structures were still being improved and developed. In 2003, Shen et al., of the University of Florida, developed a three-axis automatic scatterometer–TAAS as shown in Figure 14. The test system consisted of a high-precision goniometer platform, a laser light source, and a large dynamic range detector. The light source was manually controlled, and provided three output wavelengths, of 635 nm, 785 nm, and 1550 nm. The sample was controlled by stage 1 and stage 3 (as shown in Figure 14) to achieve different angles of incidence from the light source to the sample surface, and the divergence angle of the light source was less than 0.22 mrad. The detector was controlled by stage 2 and stage 3 (as shown in Figure 14) moves within the hemisphere on the horizontal plane to gather 3D BRDF data (incident zenith angle, reflection azimuth, and reflected zenith angle) over adjustable ranges of the zenith angle of the light source of 0°~88°, the reflected zenith angle of 0°~88°, and the reflection azimuth of 0°~180°. The system could measure the characteristics of the 1/4 space reflection. It was used to measure the BRDF data on single crystal wafers of different roughnesses, and the results were compared with those of the U.S. National Institute of Standards and Technology to verify the accuracy of the TAAS scatterometer. The authors concluded that the alignment relationship among the light source, sample, detector, and stray light influence the results [38].

**Figure 15:**
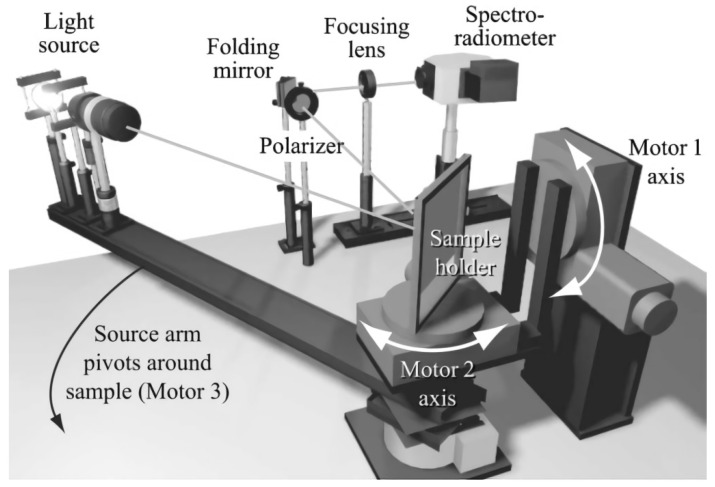
Three-axis automatic goniometer. Reprinted with permission [40]. Copyright SPIE, 2006.

To obtain the properties of the color and reflection of the material surface using computer rendering techniques, Li et al., of Cornell University, extended the available light source to the entire visible band based on an automatic triaxial goniometer, and designed a BRDF detection system based on the spectroradiometer, as shown in Figure 15. The instrument was designed to test multi-wavelength BRDF data on different material surfaces and improve the physical fidelity of computer graphics rendering. By moving the light source and the sample through the mechanical device (source arm pivoted around the sample, Motor 3) and two rotating axis structure (Motor 1, Motor 2), respectively, a wide range of incident angles of the light source relative to the sample was achieved, the moving angle resolution of the sample and light source is 0.1° and 0.13°, respectively. In order to receive the scattering signal from the wide band of the sample and the simultaneously measurement of multiple wavelengths, the detector consisted of a folded reflector, focusing mirror and spectroradiometer. The BRDFs of metallic silver paint and bright-yellow paint were measured using this system, and the former were used for rendering a 3D model of the realistic image of a car. The important application of multispectral BRDF data in model rendering was verified [39,40].

**Figure 16:**
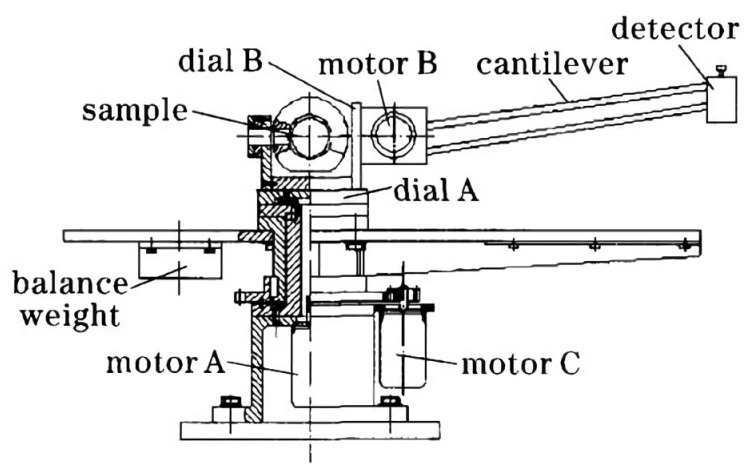
Multi-spectral BRDF measurement system. Reprinted with permission [41]. Copyright OSA, 2007.

To enable multi-spectral BRDF measurements in the hemispheric spatial range, Zhao et al., of the Harbin Institute of Technology, designed a system in 2007, as shown in Figure 16. The light source of the system was extended from the visible band to the mid-infrared band, and it was designed with an adjustable angle device (motor A, dial A) and a detector rotation module (motor B, motor C, and dial B). The sample was placed on a test bench with three degrees of freedom, and the sample rotation module was driven by motor A to rotate within ±90° to change the azimuth angles and zenith angles of the incident light on the sample surface. The mechanical arm of the detector could be moved horizontally and vertically, which is suitable for BRDF measurements at different wavelengths. The relative motion between the sample and the detector enabled the measurement of BRDF data on the surface of the sample in most hemispheres within reflection zenith angles of -55° to +55° and the reflection azimuth angles of ±180°. Sources of uncertainty in the system were also analysed, including the signal-to-noise ratio, and errors in electronic nonlinearity, the reception angle, total scattering zenith angle, and scattering reception zenith angle [41].

**Figure 17:**
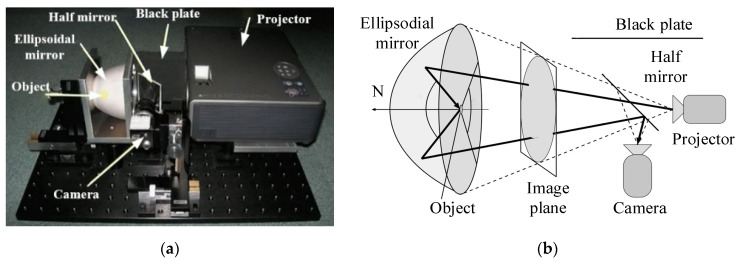
Schematic diagram of device for BRDF fast measurement and optical path based on ellipsoidal mirror. (**a**) Measurement system physical diagram; (**b**) Measurement system schematic diagram. Reprinted with permission [48]. Copyright IEEE, 2007.

As conventional measurements based on mechanically driven structures continue to evolve, their measurement time remains a prominent drawback. The BRDF distribution of a sample in hemispheric space, assuming measurements at an interval of 10° between the zenith and the azimuth angles, can be determined for up to 100,000 measurements and takes about 60 h [42]. The fast BRDF measurement technique based on aspheric optical properties continues to be developed to improve measurement efficiency [43,44,45,46,47]. A fast BRDF measurement device based on an ellipsoidal reflector and a projector was proposed by Mukaigawa et al., of Osaka University, in 2007, as shown in Figure 17. The system includes a projector, a camera, an ellipsoidal mirror, and a half-reflective half-lens. The projector was placed at the first focus of the ellipsoidal mirror as the light source, and light emitted by it was reflected by the ellipsoidal mirror to illuminate the sample at the second focal point of the ellipsoidal mirror in hemispheric space. Then, light was reflected from the sample to the ellipsoidal mirror once again. Finally, it entered the camera for imaging, and yielded the BRDFs of the spatial distributions of most hemispheres on the sample surface. The system can significantly reduce the measurement time without a mechanical drive, but the uncertainty of the source uniformity of this system and the ellipsoidal mirror had an open structure at the edge of the long axis, resulting in the final measured BRDF data being missing, and, limited by the detection camera, this system could not achieve the simultaneous measurement of specular reflection and diffuse reflection, so this system only provides a theoretical model of fast BRDF measurement for reducing the measurement time [48].

**Figure 18:**
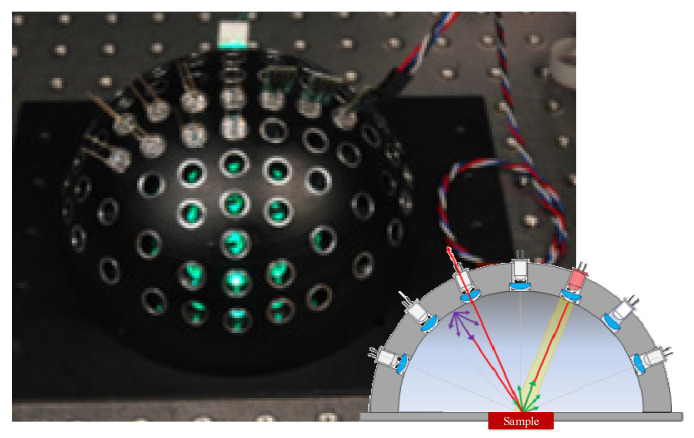
Diagram of LED-based BRDF measurement device. Reprinted with permission [49]. Copyright IEEE, 2008.

In addition to the aspheric fast measurement system, a multi-detector-based fast-measurement device was proposed in 2008 by Mosh et al. The test system was based on a spherical array, and contained only photodiodes (LEDs). The BRDF measurement device is shown in Figure 18. The system used the properties of LEDs as both light emitters and light detectors. When an LED in each array was used as the light source, the other LEDs were used as detectors. The scattered light emitted by the illumination LEDs after irradiating the sample surface was received by the other LEDs that acted as detectors in the hemispherical space array, thus enabling BRDF measurements. Owing to a lack of moving equipment in the structure, the measurement system could achieve fast measurements while remaining stable and compact, and could obtain multi-spectral BRDF measurements without being limited by the assumption of anisotropy of the sample surface. This allowed for measurements in hemispheric space without their being affected by occlusion. However, the linear array of LEDs had a low angular resolution due to limitations of the mechanical structure, and it was not possible for them to act as both a source of emission and a detector. This method, thus, cannot accurately measure backscattering [49].

**Figure 19:**
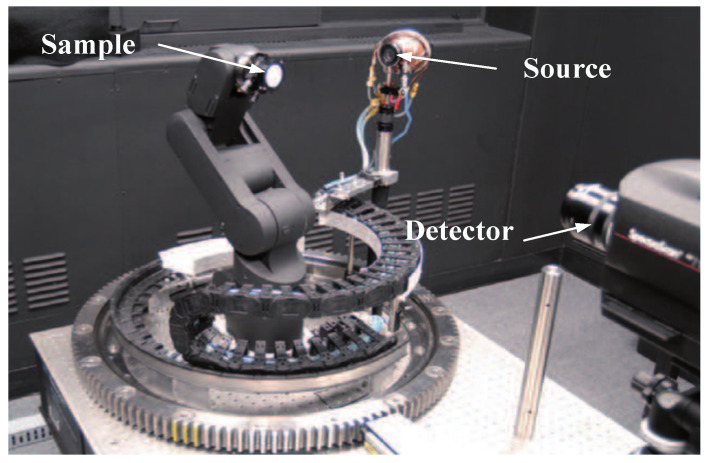
Robot-based BRDF measurement device. Reprinted with permission [50]. Copyright OSA, 2009.

With the development of robotics, Rejean et al. designed a robot-based BRDF testing device in 2009, as shown in Figure 19. The light source was a tungsten halogen lamp with a wavelength ranging from 250 nm to 1700 nm. The angle rotation device featured a circular guide and a five-axis robot arm in the centre. It could move the sample in two dimensions by rotating the arm and then used the light source to measure the three dimensions of the BRDF (incident zenith angle, incident azimuth angle, and reflected zenith angle). The two detectors used could measure wavelengths in ranges of 380–780 nm and 380–1068 nm, respectively. The system was compact as a commercial measurement device but could not achieve hemispheric spatial BRDF measurements. Moreover, the closer the detector-to-sample distance was, the lower was the resolution and the larger were the measurement errors [50].

**Figure 20:**
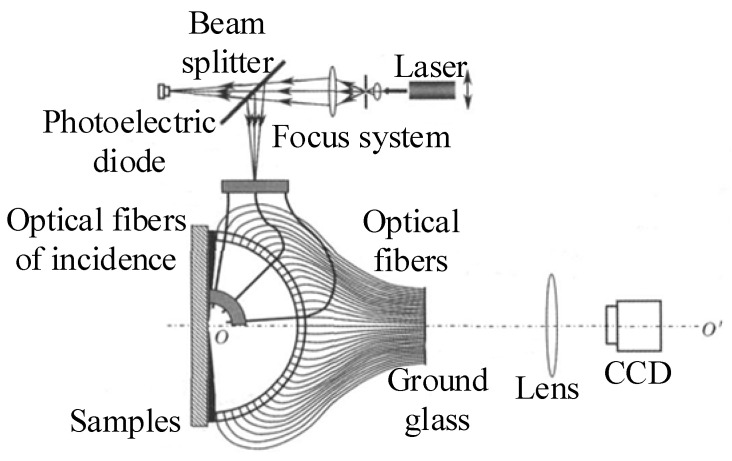
BRDF measurement device based on hemispheric spatial fiber array. Reprinted with permission [51]. Copyright Chinese Laser Press, 2009.

Following the validation of the multi-detector BRDF proposed by Mosh et al. in 2008, a BRDF measurement system based on a hemispheric spatial ray array was proposed by Ren and Zhao at Northwestern Polytechnical University in 2009. The measurement device is shown in Figure 20. Light reflected from the surface of the object at the centre of the sphere was received by the optical fiber, and processed by the CCD. The irradiated laser beam was transmitted to the point to be measured on the surface of the object using optical fibers, and incident angle of the beam could be changed by changing the bending angle of the fiber. The device could measure the zenith angle in the range of 0°~85° and the azimuth angle in the range of 0°~360°. With a fixed angle of incidence of the light source, the time required to acquire the distribution of reflection on the sample surface depended on the exposure time of the CCD and the time needed for data processing. The main advantage of this measurement system was the use of hemispheric spatial fiber array-based detection that can help avoid complex mechanical translation and rotation mechanisms, and realise a more compact structure while improving the measurement efficiency [51].

**Figure 21:**
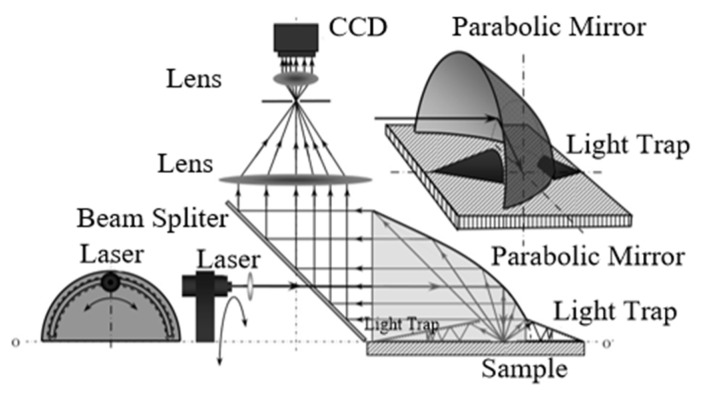
Device for fast BRDF measurement based on semi-parabolic reflector. Reprinted with permission [53]. Copyright SPIE, 2009.

Ren et al. also proposed a method for the quick measurement of the BRDF based on a semi-parabolic emission mirror. A schematic diagram of the test setup is shown in Figure 21. The device included a semi-parabolic mirror, a movable light source, a beam-splitting prism, and a faceted detection CCD. The sample was located on the axial cross-section of the semi-parabolic mirror, and could be rotated and moved in the horizontal plane. The light source was a 650 nm laser mounted on a mechanical structure that could rotate around the vertical axis to ensure incidence on the sample surface in the range of -90° to +90°. By using the optical properties of the paraboloid and beam splitter, the system was able to measure the BRDF distribution in a quarter of the space at a time, and could rotate the sample by 180° to measure another quarter of the space. The measurement process only took a few minutes [52,53].

**Figure 22:**
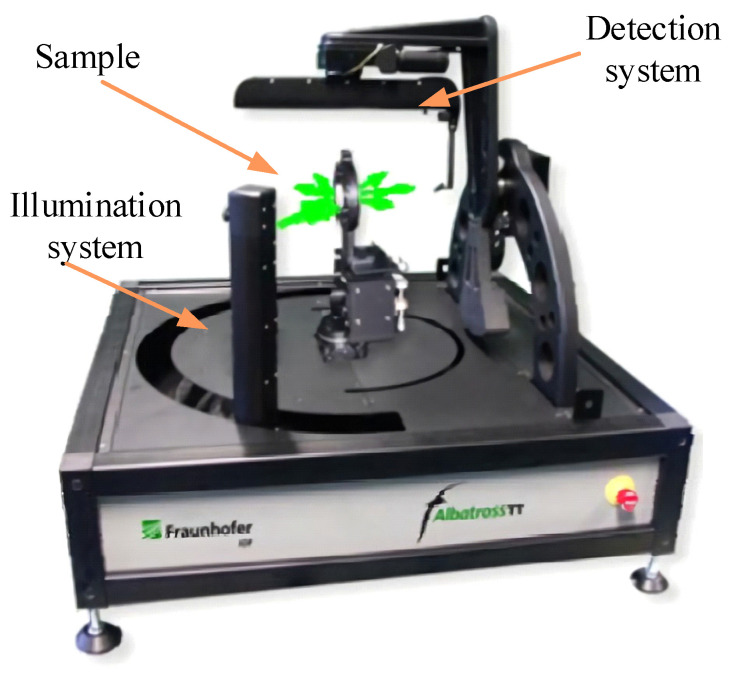
ALBATROSS-TT scatterometer. Reprinted with permission [54]. Copyright OSA, 2011.

Alexander et al., of the Fraunhofer Institute for Applied Optics and Precision Engineering, developed the ALBATROSS-TT scatterometer in 2010, as shown in Figure 22. The illumination system contained a waveplate that enabled circularly polarized light, S-polarized light or P-polarized light, to shift within ±90° of the hemispheric trajectory in the horizontal plane, and a detector that could move in this trajectory to measure BRDF data on the spatial distribution of the hemisphere. All motion mechanisms were precisely positioned above 0.01°, and could be maintained in a fixed position during measurements under S- or P-polarized light. Owing to the large size of the structure, it could measure BRDFs over a wide dynamic range using ultra-polished transparent substrates on various types of rough surfaces. The system combined polarization with conventional BRDF measurements to add a layer of polarization data to the BRDF measurements [54].

**Figure 23:**
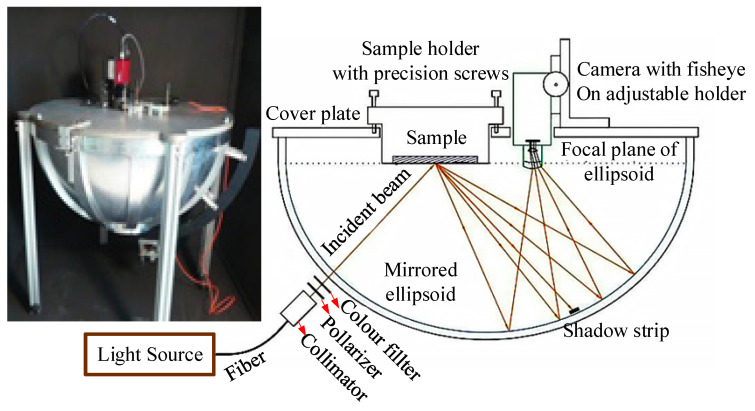
Device to quickly measure the BRDF designed using an ellipsoidal mirror. Reprinted with permission [55] Copyright Elsevier, 2013.

Based on the properties of parabolic reflection for fast BRDF measurements, a specular reflection function analyser based on an ellipsoidal reflector was designed by Meyen et al., of the German Aerospace Design Center, in 2014, as shown in Figure 23. The source of light was fiber-coupled white light that could be made to orbit in ellipsoidal trajectory to irradiate the sample zenith at angles in the range of 6.5°~40°. A filter and polarizer were placed in front of the light source for multi-spectrum measurements in different directions of polarization. The light source was incident on the sample surface at the first focal point of the ellipsoid, light scattered from the sample surface was reflected by the ellipsoid, and entered the CCD camera through the 180° fisheye lens at the second focal point of the ellipsoid. The half-ellipsoid containing the BRDF information of the sample surface was imaged by the CCD. The system can quickly measure the spatial distribution of the BRDF on a hemispheric sample surface, but the ellipsoid in the system is a critical device in which the direction of the incident light and the reception of the scattered light were dependent. A high machining accuracy was thus required, and it was challenging to guarantee a suitable cost and accuracy of machining for such a large, closed ellipsoidal mirror [55].

**Figure 24:**
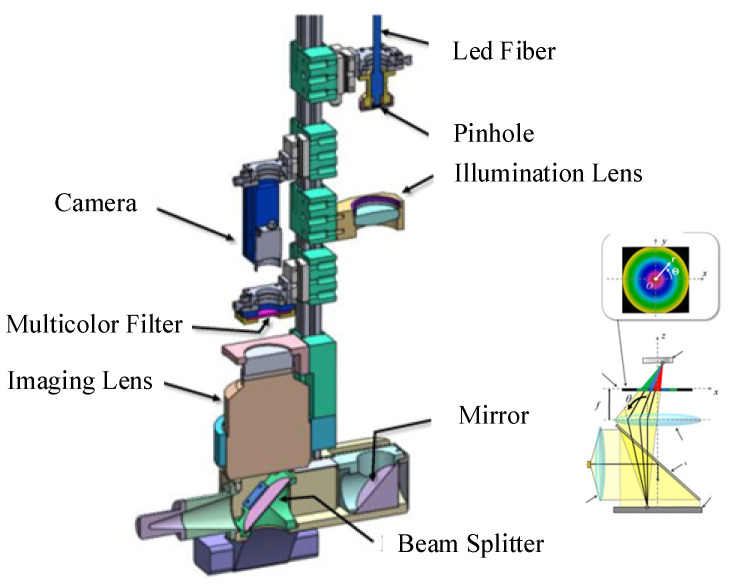
One-shot BRDF measurement device. Reprinted with permission [63] Copyright Springer 2021.

Traditional BRDF measurement technology is becoming increasingly sophisticated. At present, it is known that the BRDF measurement device with the best angular resolution is the Condor device proposed by LNE-CNAM in 2015, with an angular resolution of 0.022° [56,57,58]. The SOC series BRDF test device developed by US SOC (Surface Optics Corporation) is one of the typical commercial device that has achieved the establishment of a large BRDF database [59]. In 2016, Bother et al. combined Fourier optics with the BRDF to measure the spectral emission of displays [60]. In 2019, Luo et al. designed a set of optical paths to measure the surface tension and contact angle of liquids by means of light scattering, and achieved stable contact-free BRDF measurements of liquids using optical methods [61]. In 2021, Ohno et al. proposed a system to quickly measure the BRDF based on multi-color filters and imaging techniques, as shown in Figure 24. The system consisted of a parallel-light illumination system and an imaging optical system. The latter included an LED and a collimating lens. Collimated light was incident on the beam splitter and was reflected to the sample surface; then, light reflected from the sample surface passed through the beam splitter and was imaged on the CCD via the lenses. The colors of light in different directions after passing through the multi-color filter were different, and the color component ratio obtained from the image sensor was used to deduce the surface BRDF distribution. This was used to identify differences in the surface properties of different materials. The reconstruction of the axisymmetric micro-target surface based on data obtained from the one-shot BRDF imaging system has laid the foundation for applications of BRDF data to surface inversion [62,63]. The parameters of measurement instruments discussed in this paper are summarized in Table 1.

## 3. Trend of Development of BRDF Measurement Devices

### 3.1. Summary of Development Status

**Figure 25:**
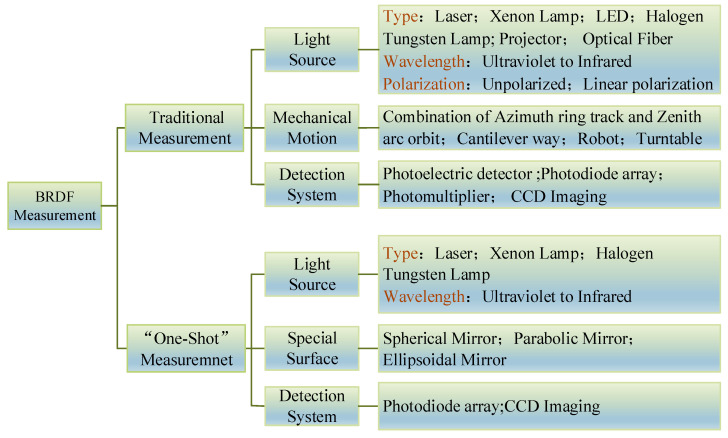
Classification of BRDF measurement devices and system components.

The initial structure used for BRDF measurement was simple and rough [64,65,66,67,68]. It was later automated to improve the accuracy and stability of the measurements. Further advances have included the enrichment of light sources [33,39,40,41,69,70] (multi-spectral, and polarization), new detectors (photoelectric detection, and CCD imaging), and the emergence of devices for fast measurement [43,44,45,46,47,48,55,62]. BRDF measurement devices are developing with the aims of being able to handle large amounts of data, and having higher accuracy, higher efficiency, and greater stability. In accordance with the history of BRDF measurements, the measurement devices can be divided into those for “traditional measurement” and “fast measurement,” as shown in Figure 25.

Traditional measurement devices measure the sample by using a mechanical structure to rotate the detector and the light source around it at a certain spatial angle. The most common structural form of such a device features a combination of the zenith of the motion structure, the azimuthal circular track [32,71,72,73,74,75,76], and a cantilever structure forms [38,41,77,78,79,80,81,82,83] for BRDF measurements. With the development of robotics, the advantages of automated measurements have been expanded by combining robots with BRDF measurement devices [44,84].

Devices capable of fast measurement are used to determine the reflective properties of the sample in hemispheric space in one shot, with the aid of the optical properties of special surfaces, special optical devices, and optical imaging techniques [34,37,43,44,45,46,47,48,52,53,60,61,62,63], or by increasing the area of the detector [49,51,85]. The measurement device uses a special surface reflector to eliminate part of the mechanical motion or increase the area of detection to measure the characteristics of reflection of the sample in hemispheric space at once. The device for fast measurement can use optics to consider the imaging device as an array of detectors to capture multiple reflections in one snapshot. This approach improves the stability of the measurement system, and, most importantly, the efficiency of the measurement. The measurement data that would take hours or even tens of hours to obtain with a traditional measuring device can be obtained in seconds or minutes with a fast measurement device.

### 3.2. Current Problems and Development Trends

A review of measurement devices reveals the following problems with current BRDF measurements:

(a) Unachievable of time-varying BRDF detection for traditional measurement;

Traditional devices perform measurements in a point-by-point manner using mechanical motion in the hemispheric space of the BRDF distribution, and even sparse sampling requires tens of hours regardless of the form of mechanical motion. Moreover, during the measurement process, the relative positional accuracy of the light source, sample, and detector has a significant influence on the measurement results, and mechanical motion introduces large instability to the measurement system. Moreover, the instability of the light source during measurement can cause intuitive errors in the BRDF data. For special samples, e.g., oxidation on the surface of objects at high temperatures or on liquids with dynamic scattered light, traditional measuring devices cannot provide BRDF measurements in variable environments over long testing periods.

(b) Lack of devices for fast measurement;

The devices for fast measurements can overcome the problems of instability and low measurement efficiency in traditional measurement devices while avoiding errors due to power fluctuations in the light source and variations in the sensitivity of the detector. They can also significantly reduce the acquisition time and capture multiple reflected light signals in the sample hemisphere space in one snapshot. However, they are limited by their mechanical structure, which can lead to missing BRDF data in a fixed direction in the hemisphere space, and the resolution of their optical system is lower than that of the traditional mechanical structure. Most importantly, the reflection outside the specular reflection area of the “one-shot” measurement is weak, and the system has a low signal-to-noise ratio, so a highly sensitive, high-precision, hyperspectral detector with a large dynamic range is needed.

(c) Lack of means to fuse and reconstruct BRDF data;

The ultimate goal of the distribution of BRDF measurements in a hemisphere is to fuse them with multi-angle information on the scattered light field to obtain the characteristics of the target, and then to invert them. However, no study to date has examined the means of fusion and reconstruction of information on the scattered light field.

(d) Incomplete analysis of factors affecting BRDF data.

The results of BRDF measurements are affected by many factors, including but not limited to the wavelength of light, angle of incidence, surface morphology of the target object, and temperature. Some studies have analysed the wavelength, angle of incidence, and observation angle but no systematic research has been devoted to the other factors.

In summary, a method to quickly measure the BRDF is needed that can simultaneously measure the multi-angle light field without requiring moving mechanical parts. It needs to also be at least as accurate as traditional measurement devices.

## 4. Conclusions

As an important means of describing the distribution of spatial optical properties on the surface of an object, the BRDF has been widely used in many fundamental and prospective research fields. In recent years, major work on the BRDF has focused on the development and application of measurement devices along two directions. This study showed, through a review of the entire developmental history of BRDF measurement devices, that the two major types of measurement devices used have their respective advantages and limitations. The foundation of the use of BRDF data is their accurate measurement, because of which research in the area is focusing on developing measurement devices with increasingly higher precision, efficiency, and stability. This can provide the basis for developing a rich database of BRDF measurements to meet the demands of many fields. Moreover, factors affecting the BRDF data and their applications need to be studied in more detail.

## Figures and Tables

**Table 1:** Summary of the technical parameters of the BRDF measurement device.

Research Institute	Data Range	Resolution	Measurement Range $(\theta_{i},$ $\varphi_{i}$ $,\theta_{r}$ $,\varphi_{r})$	Measurement Mode
University of Minnesota (Figure 6)	Anisotropy and isotropy	Sampling interval: 10°	$\theta_{i}$: 10–87° $\theta_{r}$: 0–89°	Traditional measurement
Ariozona Center for Optics Sciences (Figure 7)	Anisotropy and isotropy	Sampling interval: 10°	$\theta_{i}$: 0–60° $\theta_{r}$: −40–80°	Traditional measurement
University of Colorado (Figure 8)	Anisotropy and isotropy	Minimum sampling interval: 0.5°	$\theta_{i}$: ±86.5° $\varphi_{i}$: 0–360° $\theta_{r}$: 0–78.3° $\varphi_{r}$: 0–360°	Traditional measurement
Houston Aviation (Figure 9)	Anisotropy and isotropy	Minimum sampling interval: 0.1°	$\theta_{i}$: 5–78° $\theta_{r}$: 0–180° $\varphi_{r}$: 0–180°	Traditional measurement
Lawrence Berkeley Laboratories (Figure 10)	Anisotropy and isotropy	Minimum sampling interval: 5°	$\theta_{i}$: 0–60° $\varphi_{i}$: 0–90° $\theta_{r}$: −90–90°	Fast measurement
Cornell University (Figure 11)	Isotropy	Sampling interval: 5°	$\theta_{i}$: 0–85° $\theta_{r}$: 0–85°	Traditional measurement
University of \Miami (Figure 12)	Anisotropy and isotropy	Sampling interval: 5°	$\theta_{i}$: 5–65° $\varphi_{i}$: −180–180° $\theta_{r}$: 5–65° $\varphi_{r}$: −180–180°	Fast measurement
Rutgers University (Figure 13)	Anisotropy and isotropy	--	$\theta_{i}$: −36.5–22.8° $\theta_{r}$: −36.8–22.8° Reflected azimuth angle: 0–22.8°	Fast measurement
University of Florida (Figure 14)	Anisotropy and isotropy	Minimum sampling interval: 0.45°	$\theta_{i}$: 0–88° $\theta_{r}$: 0–88° $\varphi_{r}$: 0–180°	Traditional measurement
Cornell University (Figure 15)	Isotropy	Minimum sampling interval: 0.1°	$\theta_{i}$: −85–85° $\varphi_{i}$: −85–85° $\theta_{r}$: −85–85° $\varphi_{r}$: −85–85°	Traditional measurement
Harbin Institute of Technology (Figure 16)	Anisotropy and isotropy	Minimum sampling interval: 0.036°	$\theta_{i}$: −90–90° $\varphi_{i}$: −90–90° $\theta_{r}$: −55–55° $\varphi_{r}$: −180–180°	Traditional measurement
Osaka University (Figure 17)	Anisotropy and isotropy	Sampling interval: 1°	$\theta_{i}$: 0.5–90°; −0.5–(−90°) $\varphi_{i}$: 27–180°; −27–(−180°) $\theta_{r}$: 0.5–90°; −0.5–(−90°) $\varphi_{r}$: 27–180°; −27–(−180°)	Fast measurement
Microsoft Research Asia (Figure 18)	Anisotropy and isotropy	Minimum sampling interval: 30°	$\theta_{i}$: −90–90° $\varphi_{i}$: −180–180° $\theta_{r}$: −90–90° $\varphi_{r}$: −180–180°	Fast measurement
Institute for National Measurement Standards (Figure 19)	Anisotropy and isotropy	Minimum sampling interval: 0.125°	$\theta_{i}$: −90–90° $\theta_{r}$: −90–90° $\varphi_{r}$: −180–180°	Traditional measurement
Northwestern Polytechnical University (Figure 20)	Anisotropy and isotropy	Minimum sampling interval: 3°	$\theta_{i}$: 5°, 45°, 80° $\theta_{r}$: 0–85° $\varphi_{r}$: −180–180°	Fast measurement
Northwestern Polytechnical University (Figure 21)	Anisotropy and isotropy	--	$\theta_{i}$: −90–90° $\varphi_{i}$: −180–180° $\theta_{r}$: −90–90° $\varphi_{r}$: −180–180°	Fast measurement
Fraunhofer Institute for Applied Optics and Precision Engineering (Figure 22)	Anisotropy and isotropy	Minimum sampling interval: 0.1°	$\theta_{i}$: −90–90° $\theta_{r}$: −90–90° $\varphi_{r}$: −180–180°	Traditional measurement
German Aerospace Design Center (Figure 23)	Anisotropy and isotropy	--	$\theta_{i}$: 6.5–45° $\theta_{r}$: −90–90° $\varphi_{r}$: −180–180°	Fast measurement
Toshiba Corporation (Figure 24)	Anisotropy and isotropy	Sampling interval: 0.25°	--	Fast measurement

## Data Availability

Not applicable.
